# Interictal widespread pressure hyperalgesia and aura: associations with vestibular migraine in a cross-sectional study

**DOI:** 10.3389/fneur.2024.1405590

**Published:** 2024-07-03

**Authors:** Toshihide Toriyama, Yoshiki Hanaoka, Tetsuyoshi Horiuchi

**Affiliations:** ^1^Toriyama Clinic, Komoro, Japan; ^2^Department of Neurosurgery, Shinshu University School of Medicine, Matsumoto, Japan

**Keywords:** migraine, vestibular migraine, allodynia, central sensitization, hyperalgesia, interictal

## Abstract

**Background:**

Patients with vestibular migraine (VM) exhibit higher levels of central sensitization and share similar disorder characteristics with migraine with vestibular symptoms (MwVS), except in terms of disability. These patients experience fluctuating mechanical pain thresholds and persistent vestibular symptoms even without a migraine attack.

**Objective:**

This study aimed to investigate whether interictal allodynia or hyperalgesia can differentiate between VM, MwVS, and migraine only.

**Methods:**

We conducted a cross-sectional study of patients with episodic migraine aged between 18 and 65 years, categorized into three groups. A questionnaire was used to collect and compare demographic and clinical variables. Interictal widespread pressure hyperalgesia (IWPH) was evaluated using the Manual Tender Point Survey. Patients with tender point counts ≥7 were classified as having IWPH.

**Results:**

The study included 163 patients: 31 with VM, 54 with MwVS, and 78 with migraine without vestibular symptoms (migraine only). We found that aura (*p* = 0.042, odds ratio 3.50, 95% confidence interval 1.26–10.4), tender point count (p < 0.001, d = 0.889, median difference = 2), and IWPH (*p* = 0.002, odds ratio 5.3, 95% confidence interval 1.80–17.2) were significantly associated with VM compared to MwVS. Aura and IWPH were significantly associated with VM. However, there were no significant associations observed for interictal allodynia or hyperalgesia between the other two groups.

**Conclusion:**

IWPH and aura are associated with VM, indicating their potential roles in its pathogenesis. These findings may contribute to the differential diagnosis and management of migraine, potentially leading to targeted treatment strategies.

## Introduction

Vestibular migraine (VM) is a leading cause of episodic vertigo and dizziness (1–4); however, it was not well-known until its inclusion in the new classification by the ICHD-beta version in 2013 (5). There was no consensus regarding the diagnostic criteria for VM among the patients included in the studies, resulting in a limited understanding of its characteristics and pathophysiology (6, 7). VM is a subtype of migraine marked by hypersensitivity to self-motion (8) and heightened sensitivity in vestibular pathways (9). Cutaneous allodynia (CA) and hyperalgesia are common in migraine and are manifestations of central sensitization (10–16). Migraine with vestibular symptoms not entirely fitting VM criteria (MwVS) is associated with more CA than migraine without (17, 18). Our previous study suggests that the pathogenesis of VM might be linked to thalamic sensitization, as patients with VM exhibit a stronger association with all CA subtypes compared to those with non-vestibular episodic migraine (17, 19). Mechanical pain thresholds in patients with migraine fluctuate during the migraine cycle (20), and they may experience persistent central sensitization leading to vestibular symptoms without headaches (21, 22).

We hypothesized that interictal allodynia and hyperalgesia may help distinguish VM, MwVS, and migraine only (MO) (14, 15, 23, 24). This study aimed to compare the demographic and clinical characteristics of VM, MwVS, and MO during the interictal phase, explore associations between VM and MwVS, and identify significant risk factors related to VM. To our knowledge, this is the first study to examine the difference in interictal allodynia and hyperalgesia prevalence between patients with and without VM.

## Methods

### Design and setting

Patients with migraine were recruited for a cross-sectional survey from January 2018 to March 2021 at Toriyama Clinic, a local primary and secondary headache clinic in Komoro City, Nagano Prefecture, Japan, serving a target population of approximately 100,000. Each participant underwent a structured interview and comprehensive clinical assessment conducted by the first author, an experienced neurologist, to determine their eligibility based on predefined inclusion and exclusion criteria.

This is a secondary analysis of data following our original research plan. From our prior study (17), 101 of the 245 cases were interictal and are included here. The initial study did not cover all findings due to word limits. Our aim now, with a focus on interictal widespread pressure hyperalgesia (IWPH), was to expand the interictal sample size, merging new and prior cases.

### Participants

**Figure 1:**
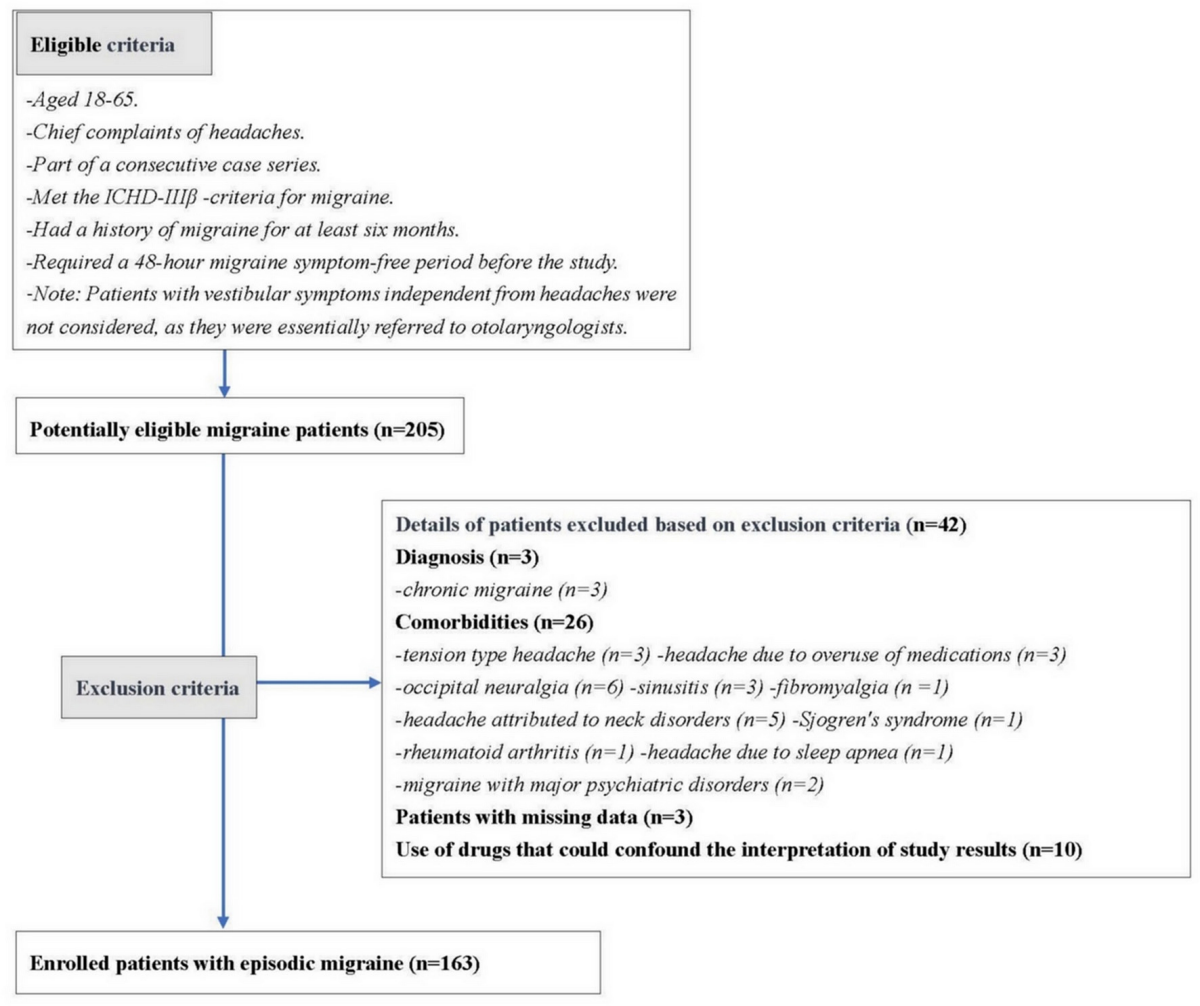
Eligibility and exclusion criteria.

Participants aged 18–65 years, with chief complaints of headaches and part of a consecutive case series, were included in this study. These individuals met the International Classification of Headache Disorders (ICHD)-IIIβ criteria for migraine and had a history of migraine for at least 6 months. In this study, the aura was limited to typical auras such as visual, sensory, and verbal types. Vertigo was not considered an aura. Additionally, to minimize the impact of acute allodynia, we required a 48-h migraine symptom-free period before the study. Patients with vestibular symptoms independent from headaches were not considered, as they were essentially referred to an otolaryngologist for specialized management. Patients with other primary or secondary headaches, specific disorders, incomplete data, and those taking medications (beta blockers, antidepressants, anticonvulsants, and calcium channel blockers)/antineuropathic pain agents (pregabalin, gabapentin, and duloxetine) that could potentially influence the results were excluded from the study (Figure 1).

### Clinical evaluation

**Figure 2:**
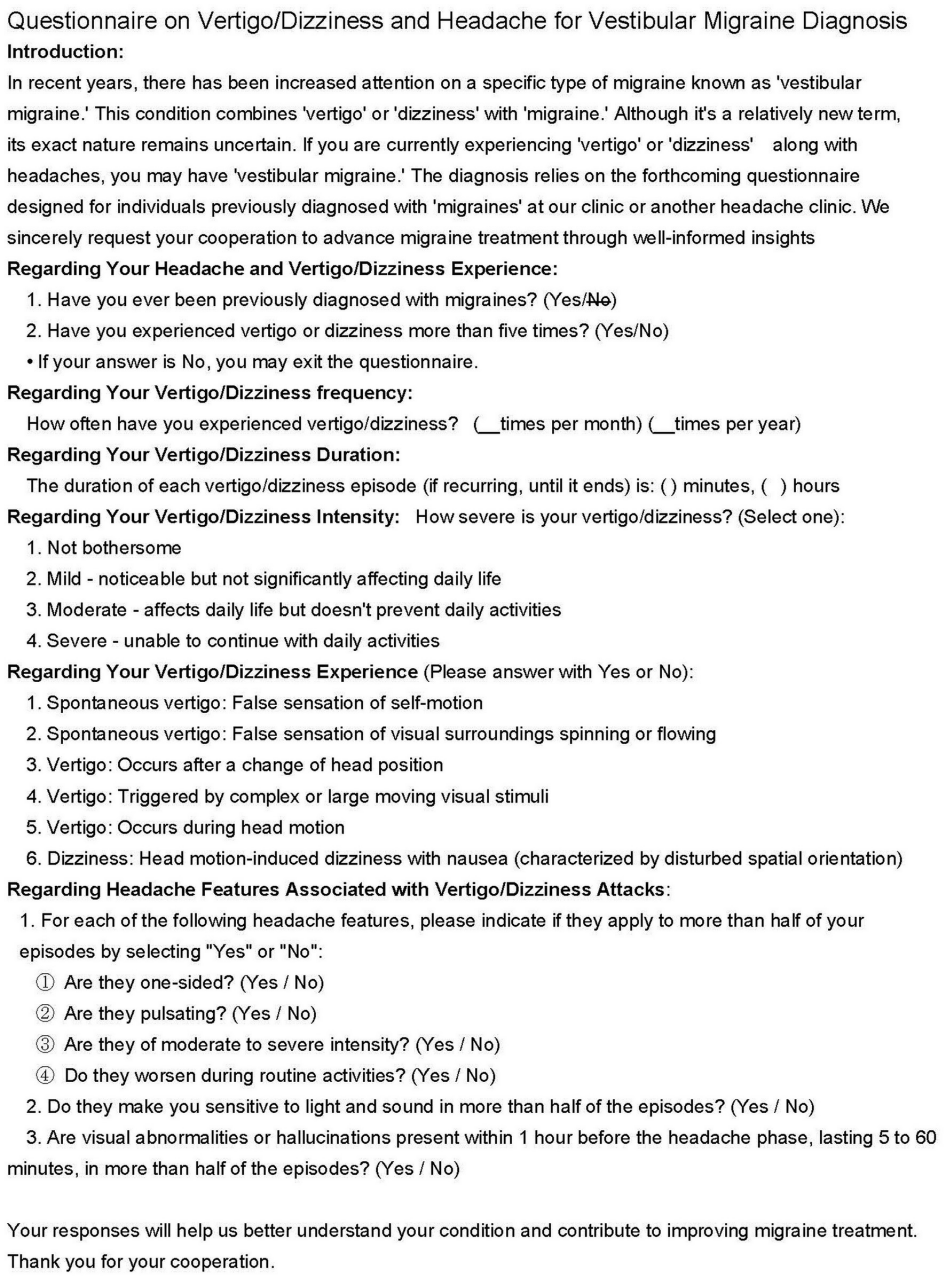
Questionnaire on vertigo for vestibular migraine.

Participants underwent evaluation based on the ICHD-IIIβ criteria, including assessment of demographic characteristics and associated symptoms, with particular focus on vestibular symptoms identified using a questionnaire (Figure 2) compliant with the International Classification of Vestibular Disorders (25). Different types of migraines—both with and without aura—may be experienced by patients over time. However, to ensure consistency and accuracy in reporting clinical characteristics, each patient was classified based on their most recent episode. Participants were then categorized into the VM, MwVS, or MO groups. Migraine-specific variables and associated symptoms were documented, along with a record of medication history.

### Measurement

**Figure 3:**
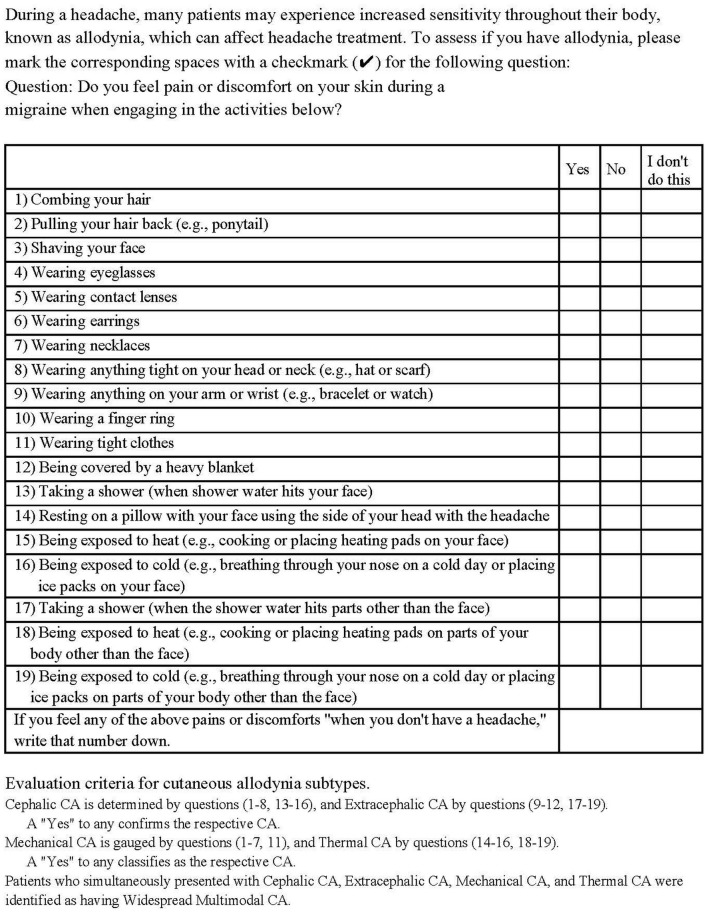
Nineteen-item allodynia questionnaire.

Figure 3 provides details of the 19-item questionnaire and evaluation criteria for cutaneous allodynia subtypes.

Headache intensity was assessed using a numerical rating scale (26), headache disability was assessed using the Headache Impact Test-6 (HIT-6) (27), depression was assessed using the Self-Rating Depression Scale (SDS) (28), and tinnitus and sleep disturbances were assessed using yes/no questions.

CA symptoms were assessed using a 19-item questionnaire adapted from Ashkenazi et al. (14) and ASC-12 (23). Patients who confirmed experiencing discomfort or pain during specific activities related to migraine were classified as allodynic if they reported **two** or more items (14). An additional **three** items by Guy et al. (29) were included to identify extracephalic CA.

Interictal CA was identified in patients who reported experiencing at least **one** allodynia symptom during headache-free periods using a questionnaire (30). Cephalic and extracephalic CA were determined based on items suggested by Guy et al. (29), with affirmative responses indicating the presence of these conditions.

Mechanical and thermal CA were assessed with specific queries, and positive responses indicated the presence of these conditions (17), following the conventions of previous surveys (13, 23). Patients exhibiting thermal, mechanical, cephalic, and extracephalic CA were identified as having widespread multimodal CA (31).

IWPH was evaluated using the Manual Tender Point Survey (MTPS) (32). Patients with a tender point count (TPC) of ≥7 were classified as having IWPH (33). In a pilot study, test-retest reliability for all assessments ranged from moderate to substantial (Table 1).

**Table 1:** Pilot study results: reliability for CA types and IWPH assessments.

**Assessment**	**n**	**Age (years)**	**Female (%)**	**Test-retest interval (days)**	**Cohen's κ**	**95% CI**	**Interpretation of κ**
Acute CA	84	40.9 ± 10.5	89.2	87.1 ± 25.4	0.57	0.29–0.84	Moderate agreement
Interictal CA	51	42.2 ± 11.7	92.2	134.5 ± 58.7	0.71	0.52–0.91	Substantial agreement
Cephalic CA	84	40.9 ± 10.5	89.2	87.1 ± 25.4	0.73	0.52–0.93	Substantial agreement
Extracephalic CA	84	40.9 ± 10.5	89.2	87.1 ± 25.4	0.59	0.41–0.76	Moderate agreement
Thermal CA	84	40.9 ± 10.5	89.2	87.1 ± 25.4	0.60	0.43–0.79	Moderate agreement
Mechanical CA	84	40.9 ± 10.5	89.2	87.1 ± 25.4	0.62	0.41–0.83	Substantial agreement
Widespread multimodal CA	84	40.9 ± 10.5	89.2	87.1 ± 25.4	0.62	0.45–0.79	Substantial agreement
IWPH assessment	84	40.9 ± 10.5	89.2	87.1 ± 25.4	0.71	0.50–0.93	Substantial agreement

CA, cutaneous allodynia; IWPH, interictal widespread pressure hyperalgesia.

### Statistical analysis

Continuous variables were presented as mean ± standard deviation or percentages. The normality of the data was assessed using the Kolmogorov–Smirnov test. One-way analysis of variance (ANOVA) was used for normally distributed data, while the Kruskal–Wallis test was employed for non-parametric distributions. Chi-squared analysis was used for categorical variables.

A multivariable logistic regression model was initially constructed in an exploratory manner, incorporating variables with *p* < 0.3 from the *post-hoc* comparison. We chose bivariate screening to detect patterns without preset constraints. Backward stepwise selection was then applied to refine the model, retaining only variables with *p* < 0.05. Statistical significance was defined as a two-tailed *p* < 0.05. Odds ratios (ORs), 95% confidence intervals (CIs), and Cohen's r for non-parametric effect size were calculated.

The sample size was determined based on available data without prior statistical power calculations. All statistical analyses were performed using EZR version 1.40 (34).

## Results

**Figure 4:**
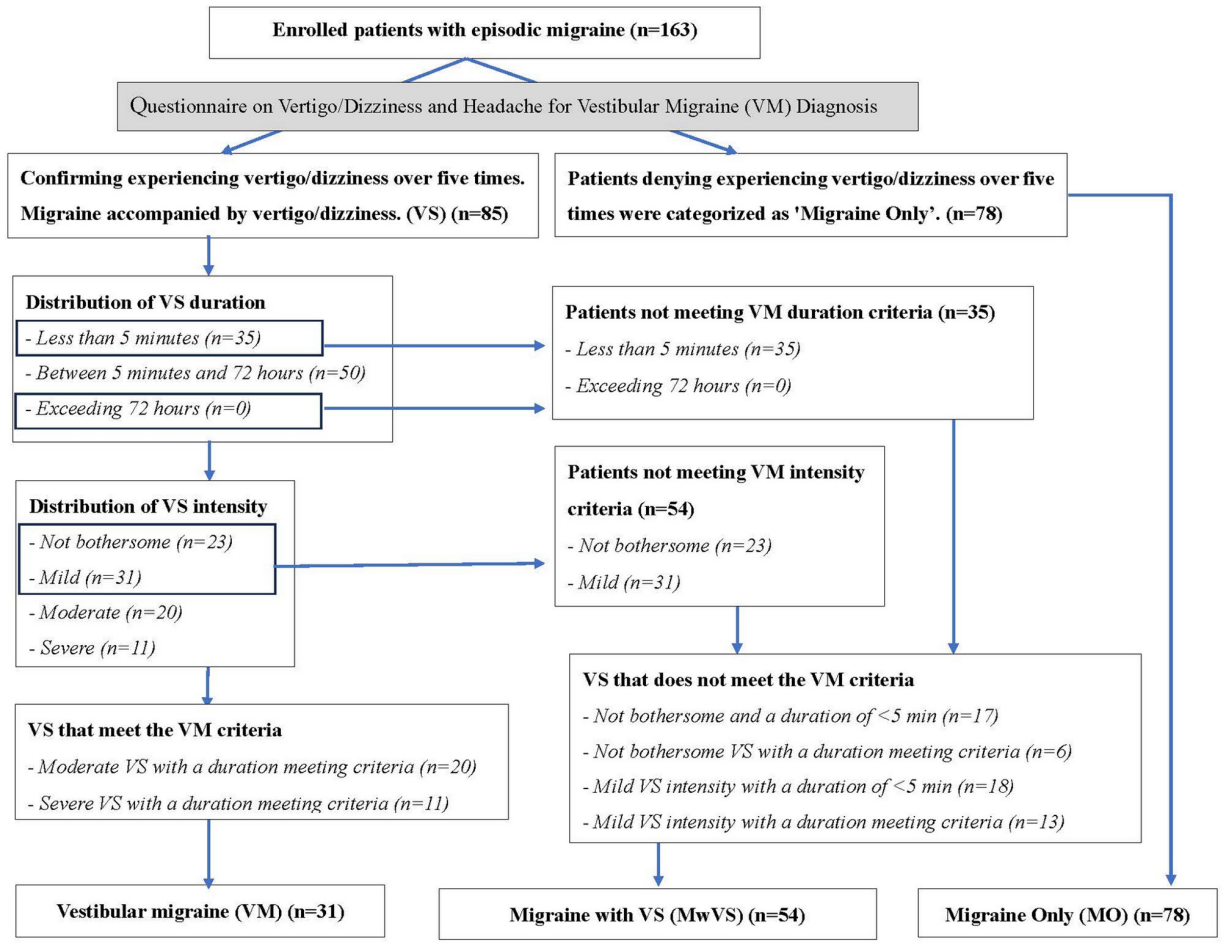
Flowchart depicting patient categorization into three groups.

A total of 205 patients with potential interictal migraine were initially recruited for the study. However, 42 participants were excluded due to comorbidities, missing data, or the use of medications that could affect the results (Figure 1). Ultimately, 163 patients with episodic migraine (mean age: 40.9 ± 11.5 years; 128 females: 78.5%) were enrolled in the study. Among these, 31 (19%), 54 (33.1%), and 78 (47.9%) patients were assigned to the VM, MwVS, and MO groups, respectively. Vestibular symptoms were reported in 85 participants. Within the MwVS group, 23 participants did not meet the duration criterion, and 31 did not meet the duration and disability criteria (Figure 4). Demographic and clinical characteristics were compared between the three groups (Table 2). The MTPS results for the three groups are presented in Table 3. Significant differences were found in the prevalence of aura, osmophobia, tinnitus, acute CA, TPC, and IWPH. However, no significant differences were found in sex, age, age at migraine onset, duration, attack frequency and duration, headache intensity, family history, nausea/vomiting, photophobia, phonophobia, depression, sleep disorders, interictal CA, or medication use (*p* > 0.05) among the three groups.

**Table 2:** Comparison of variables between VM, MwVS, and MO groups.

**Variables**	**VM**	**MwVS**	**MO**	***p*-value**	**Pairwise comparisons (** ***p*** **-value)**		
	**(*****n*** = **31)**	**(*****n*** = **54)**	**(*****n*** = **78)**		**VM- MwVS**	**VM-MO**	**MwVS-MO**
General variables							
Female sex	27 (87.1%)	45 (83.3%)	56 (71.8%)	0.123^a^	NA	NA	NA
Age, years	39.8 ± 12.8	41.5 ± 11.0	40.9 ± 11.5	0.817^b^	NA	NA	NA
Migraine-specific variables							
Migraine with aura	22 (71.0%)	22 (40.7%)	30 (38.5%)	**0.006** ^a^	***0.042*** ^c^	***0.013** ^***c***^*	p > 0.999 ^c^
Age at migraine onset (years)	21.2 ± 9.3	22.4 ± 10.6	21.2 ± 9.3	0.805^d^	NA	NA	NA
Migraine duration (years)	19.7 ± 12.1	19.1 ± 11.9	19.7 ± 11.2	0.866^d^	NA	NA	NA
Duration of headache attack (h)	15.1 ± 15.3	20.4 ± 19.8	18.8 ± 21.3	0.616^d^	NA	NA	NA
Headache frequency (attacks/month)	3.9 ± 4.3	2.7 ± 2.6	2.8 ± 2.9	0.607^d^	NA	NA	NA
Headache intensity (NRS)	7.5 ± 1.6	7.0 ± 1.7	7.3 ± 1.4	0.605^d^	NA	NA	NA
Headache disability (HIT-6)	62.5 ± 7.4	59.9 ± 7.5	60.1 ± 5.8	0.219^d^	NA	NA	NA
First-degree relative FH	18 (58.1%)	37 (68.5%)	47 (60.3%)	0.532^a^	NA	NA	NA
Migraine-associated symptoms							
Nausea/vomiting	31 (100%)	51 (94.4%)	72 (92.3%)	0.284^e^	NA	NA	NA
Photophobia	28 (90.3%)	41 (75.9%)	58 (74.4%)	0.178^e^	NA	NA	NA
Phonophobia	27 (87.1%)	42 (77.8%)	56 (74.4%)	0.254^e^	NA	NA	NA
Osmophobia	19 (61.3%)	27 (50.0%)	25 (32.1%)	***0.011** ^***a***^*	*p* > 0.999 ^c^	***0.029** ^***c***^*	0.175^c^
Depression (SDS ≥ 48)	8 (25.8%)	43 (20.4%)	12 (1.4%)	0.436^a^	NA	NA	NA
Tinnitus	14 (45.2%)	12 (22.2%)	9 (11.5%)	**0.001** ^a^	0.148 ^c^	*** < 0.001** ^***c***^*	0.477^c^
Sleep disorders	6 (19.4%)	11 (20.4%)	8 (10.3%)	0.224 ^a^	NA	NA	NA
Interictal cutaneous allodynia	7 (22.6%)	9 (16.7%)	7 (9.0%)	0.148 ^a^	NA	NA	NA
Acute cutaneous allodynia	25 (80.6%)	33 (61.1%)	41 (52.6%)	**0.026** ^a^	0.361^c^	***0.038** ^***c***^*	*p* > 0.999^c^
Medication							
Use of acute medication	14 (45.2%)	30 (55.6%)	43 (55.1%)	0.595 ^a^	NA	NA	NA
Use of triptans	19 (61.3%)	33 (61.1%)	46 (48.1%)	0.960 ^a^	NA	NA	NA
No medication	0 (0.0%)	2 (3.7%)	3 (3.8%)	0.545 ^e^	NA	NA	NA
Manual tender point survey							
TPC	8.9 ± 3.8	5.4 ± 3.9	4.97 ± 4.2	*** < 0.001** ^*b*^*	**< 0.001** ^f^	*** < 0.001** ^*f*^*	*p* > 0.999^f^
IWPH (%)	24 (77.4%)	21 (38.9%)	24 (30.8%)	**< 0.001** ^a^	**0.002** ^c^	*** < 0.001** ^*c*^*	*p* > 0.999^c^
Allodynia subtypes							
Cephalic CA	27 (87.1%)	43 (79.6%)	52 (66.7%)	0.052^a^	NA	NA	NA
Extracephalic CA	23 (74.2%)	29 (53.7%)	33 (42.3%)	**0.008** ^a^	0.306^c^	***0.016** ^***c***^*	0.798^c^
Mechanical CA	26 (83.9%)	33 (61.1%)	41 (52.6%)	***0.006** ^*a*^*	0.152 ^c^	***0.021** ^***c***^*	*p* > 0.999^c^
Thermal CA	17 (54.8%)	25 (46.3%)	36 (46.2%)	0.688^a^	NA	NA	NA
Widespread multimodal CA	16 (51.6%)	17 (31.5%)	16 (20.5%)	**0.00 6** ^a^	0.327^c^	***0.014** ^***c***^*	0.883^c^

Values represent absolute numbers with corresponding percentages or means ± standard deviations. Bold and italicized text indicate significant p-values (p < 0.05). FH, family history; HIT-6, Headache Impact Test; MO, migraine only; MwVS, migraine with vestibular symptoms not meeting vestibular migraine criteria; NA, not applicable; NRS, numeric rating scale; SDS, self-rating depression scale; VM, vestibular migraine. ^a^χ^2^-test; ^b^One-way analysis of variance; ^c^Bonferroni test; ^d^Kruskal–Wallis test; ^e^Fisher's exact test; ^f^Steel–Dwass test.

**Table 3:** Comparison of TPC and IWPH frequency using MTPS between the VM, MwVS, and MO groups.

**Variables**	**VM**	**MwVS**	**MO**	***p*-value**	**Pairwise comparisons (** ***p*** **-value)**		
	**(*****n*** = **31)**	**(*****n*** = **54)**	**(*****n*** = **78)**		**VM- MwVS**	**VM-MO**	**MwVS-MO**
TPC	8.9 ± 3.8	5.4 ± 3.9	4.97 ± 4.2	*** < 0.001** ^*b*^*	*** < 0.001** ^*f*^*	*** < 0.001** ^*f*^*	*P* > 0.999^f^
IWPH (%)	24 (77.4%)	21 (38.9%)	24 (30.8%)	*** < 0.001** ^*a*^*	**0.002** ^c^	**< 0.001** ^c^	*P* > 0.999^c^

Bold and italicized text indicates significant p-values (p < 0.05). IWPH, interictal widespread pressure hyperalgesia; MTPS, Manual Tender Point Survey; MO, migraine without vestibular symptoms; MwVS, migraine with vestibular symptoms not meeting the vestibular migraine criteria; NA, not applicable; TPC, tender point count; VM, vestibular migraine. ^a^χ^2^-test; ^b^Kruskal–Wallis test; ^c^Bonferroni test; ^f^Steel–Dwass test.

*Post-hoc* pairwise comparisons of the significant variables between the three groups revealed that the VM group had a significantly higher frequency of migraine with aura (p = 0.042, OR 3.50, 95% CI 1.26–10.39), TPC (*p* < 0.001, *r* = 0.861, median difference = 2), and IWPH prevalence (*p* = 0.002, OR 5.2, 95% CI 1.80–17.2) compared to the MwVS group. Similarly, the VM group had significantly higher frequencies of migraine with aura (OR 3.78, 95% CI 1.19–12.9), osmophobia (*p* = 0.029, OR 5.2, 95% CI 1.59–19.4), and tinnitus (*p* = 0.029, OR 5.4, 95% CI 1.39–26.4), as well as a higher prevalence of acute CA (*p* = 0.038, OR 4.3, 95% CI 1.27–16.7), TPC (*p* < 0.001, *r* = 0.868, median difference = 4) and IWPH (*p* < 0.001, OR 6.9 95% CI 2.06–26.3) compared to the MO group. No significant differences in clinical features were found between the MwVS and MO groups (Tables 2, 3).

Significant differences were observed in specific CA subtypes among the groups (Table 4). Extracephalic (*p* = 0.008), mechanical (p = 0.006), and widespread multimodal CA (*p* = 0.006) showed significant differences among the three groups. However, there were no significant differences in allodynia subtypes between the VM and MwVS groups or between the MwVS and MO groups. In comparison to the MO group, the VM group had significantly higher rates of extracephalic (*p* = 0.016, OR 8.7, 95% CI 2.03–25.1), mechanical (*p* = 0.021, OR 8.1, 95% CI 1.71–25.9), and widespread multimodal CA (*p* = 0.014, OR 14.7, 95% CI 2.89–149.13).

**Table 4:** Comparison of the frequency of CA subtypes between VM, MwVS, and MO groups.

**Allodynia subtypes**	**VM**	**MwVS**	**MO**	***p*-value**	**Pairwise comparisons (** ***p*** **-value)**		
	**(*****n*** = **31)**	**(*****n*** = **54)**	**(*****n*** = **78)**		**VM-MwVS**	**VM-MO**	**MwVS-MO**
Cephalic CA	27 (87.1%)	43 (79.6%)	52 (66.7%)	0.052^a^	NA	NA	NA
Extracephalic CA	23 (74.2%)	29 (53.7%)	33 (42.3%)	**0.008** ^a^	0.306^c^	***0.016** ^***c***^*	0.798^c^
Mechanical CA	26 (83.9%)	33 (61.1%)	41 (52.6%)	**0.006** ^a^	0.152^c^	***0.021** ^***c***^*	*p* > 0.999^c^
Thermal CA	17 (54.8%)	25 (46.3%)	36 (46.2%)	0.688^a^	NA	NA	NA
Widespread multimodal CA	16 (51.6%)	17 (31.5%)	16 (20.5%)	**0.006** ^a^	0.327^c^	***0.014** ^***c***^*	0.883^c^

Values represent absolute numbers with corresponding percentages. Bold and italicized text indicate significant p-values (p < 0.05). CA, cutaneous allodynia; VM, vestibular migraine; MwVS, migraine with vestibular symptoms not meeting the vestibular migraine criteria; MO, migraine only. ^a^χ^2^-test, ^b*c*^Bonferroni test.

In the multivariable logistic regression analysis of variables with *p* < 0.3, based on the *post-hoc* comparison of the VM and MwVS groups, aura and IWPH were found to be significantly associated with VM (*p* = 0.025, OR 3.15, 95% CI 1.15–8.6 and *p* = 0.003, OR 4.9, 95% CI 1.75–13.8, respectively) (Table 5).

**Table 5:** Multivariate logistic regression model VM-related factors in patients with migraine with vestibular symptoms.

**Variables**	**Odds ratio**	**95% IC**	***P***
Aura	3.15	1.15–8.6	0.025
IWPH	4.9	1.75–13.8	0.003

Independent variables with a p < 0.3 in the post hoc univariate analysis were introduced in the model: aura, tinnitus, mechanical cutaneous allodynia, and IWPH. CI, confidence interval; IWPH: interictal widespread pressure hyperalgesia; VM, vestibular migraine.

The data supporting the findings of this study are presented in Supplementary File 1.

## Discussion

### Main findings

This study included 163 patients who were divided into VM (19%), MwVS (33.1%), and MO (47.9%) groups. Significant differences were found between groups in aura frequency, osmophobia, tinnitus, prevalence of acute CA, allodynia subtypes, TPC, and prevalence of IWPH. The prevalence of interictal CA was low and did not differ between the groups. Patients in the VM group exhibited significantly higher TPC and a higher prevalence of interictal IWPH compared to those in the MwVS and MO groups. Multivariable logistic regression analysis indicated that aura and IWPH have a stronger association with VM than with MwVS.

### VM prevalence

Over half (52.1%) of patients with migraine experienced vestibular symptoms, which is consistent with the prevalence reported in previous research studies (51.7–61%) (18, 35, 36). The observed VM prevalence was 26.5%, surpassing the previous rates of 9–12% and 10.3% before and after the implementation of the new criteria (1, 36, 37). These findings support Calhoun et al.'s discovery of a strong correlation between migraine pain and vertigo (38), as nearly half of the participants in our study experienced vertigo or dizziness with high headache intensity. Considering the research conducted at a headache clinic that treats severe headaches, it is reasonable to speculate that this high prevalence might not reflect actual variations in the general population but could be due to selection bias from referral patterns and patient preferences.

### Differences between VM and MwVS

VM has a higher frequency of aura, TPC, and IWPH than MwVS. Based on our results, these three parameters significantly characterize VM compared to MwVS. Patients with VM exhibit a higher frequency of migraine aura, higher TPC, and greater prevalence of IWPH than those with MwVS. In multivariable logistic regression analysis, migraine aura and IWPH were independently associated with VM compared to MwVS.

In our previous study (20) of patients with both ictal and interictal migraine, no clinical differences were found between VM and MwVS except for the disability caused by possible selection bias. Thus, we believe that VM and MwVS may be on the same disease spectrum, which aligned with the findings of Abouzari et al. (19). However, this hypothesis has been challenged in this study, which suggested different pathophysiologies of aura and interictal hyperalgesia as the reason for the differences between VM and MwVS.

### Distinct features of VM: aura, TPC, and IWPH

The migraine-related factors associated with VM (aura, TPC, and IWPH) are summarized here as distinct features. The discussions of aura, TPC, and IWPH (previously in sections 4.5 Aura and VM, 4.13 TPC and VM, and 4.14 IWPH and VM) have been revised and moved here for greater conciseness without compromising key insights and findings from their previous locations.

The prevalence of migraine with aura in this study was 45.4%, higher than previously reported (12–36%) (1, 36). The higher VM prevalence may be influenced by factors like referral patterns, population differences, or regional specialty choices. Visual aura symptoms, resembling transient ischemic attacks, could direct patients to stroke clinics. Further research is needed to determine the cause. The relationship between vertigo and migraine, with or without aura, remains debated. Some studies have found an association between migraine with aura and vertigo (18), while others have reported more frequent vertigo in patients with migraine without aura (3, 36, 39–41). Recent findings challenge this and demonstrate a stronger correlation between migraine with aura and VM compared to MwVS or MO (38). Additionally, patients with migraine who experience aura are more susceptible to postural control impairments (42).

Our research, supported by logistic regression analysis, confirms a significant association between vestibular symptoms and migraine with aura, emphasizing the crucial role of aura in the onset of VM. Cutrer and Baloh proposed that the mechanism of cortical spreading depression (CSD) causes short-duration vertigo accompanied by headaches lasting from minutes to 2 h (43). Demarquay et al. (44) proposed that brainstem aura (vertigo/dizziness) is a typical migraine aura resulting from transient parieto-insular vestibular cortex dysfunction caused by CSD. These symptoms may occur before or during headache attacks, lasting between 5 min and 1 h, meeting VM duration criteria.

While we confirmed the link between aura and VM, it is crucial to note that vestibular symptoms can arise at any migraine stage, not just as an aura.

**Figure 5:**
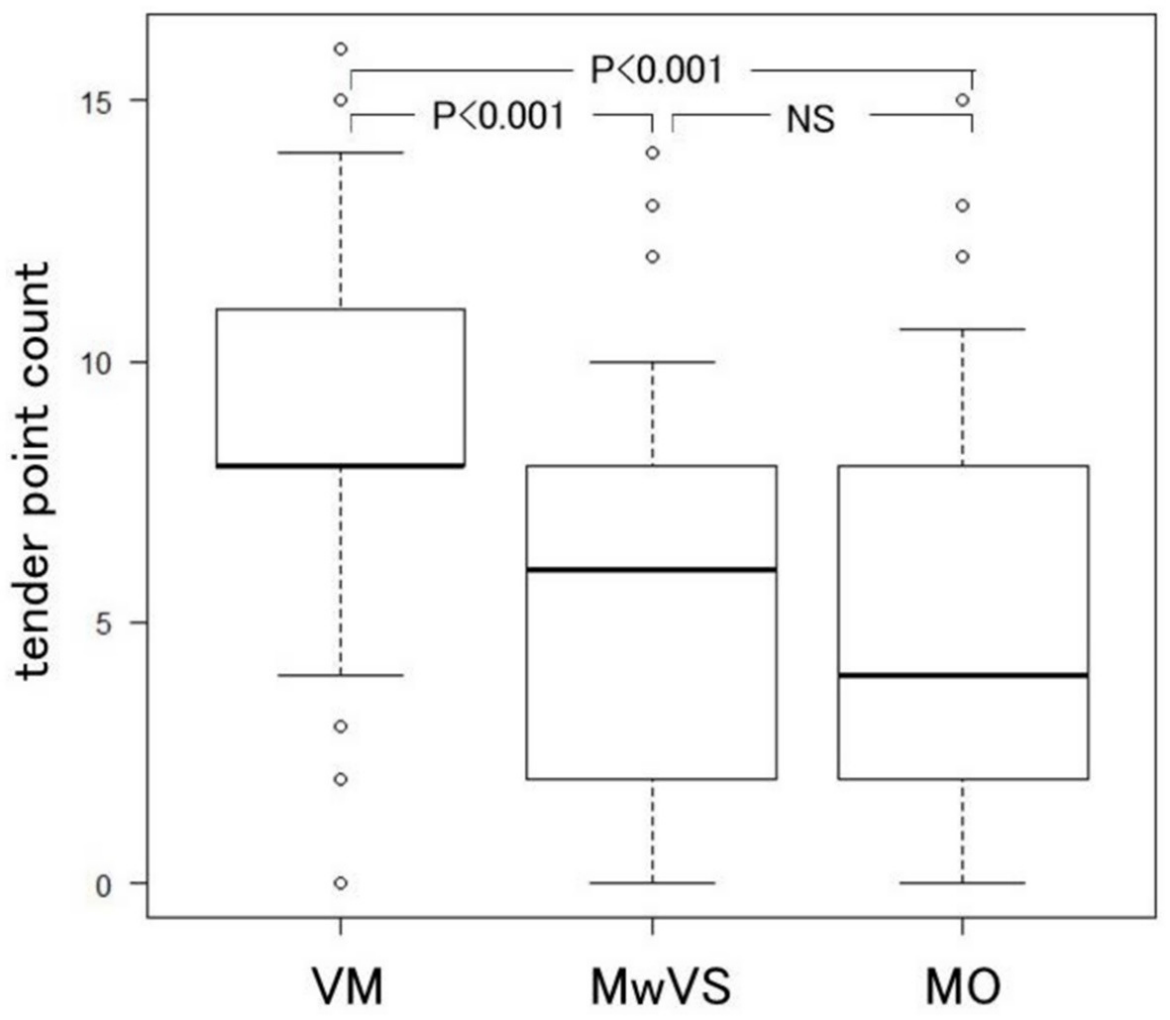
Box plot comparison of tender point counts (TPC).

*Post-hoc* pairwise comparisons revealed that VM exhibited significantly higher TPCs than MwVS or MO, indicating that VM generally had a lower pressure pain threshold (PPT) during the interictal phase (Figure 5). This finding suggests a widespread decrease in PPT, as a higher TPC corresponds to a reduced PPT measured by QST (33, 54). Therefore, TPC has the potential to differentiate VM from MwVS in migraine patients with vestibular symptoms.

**Figure 6:**
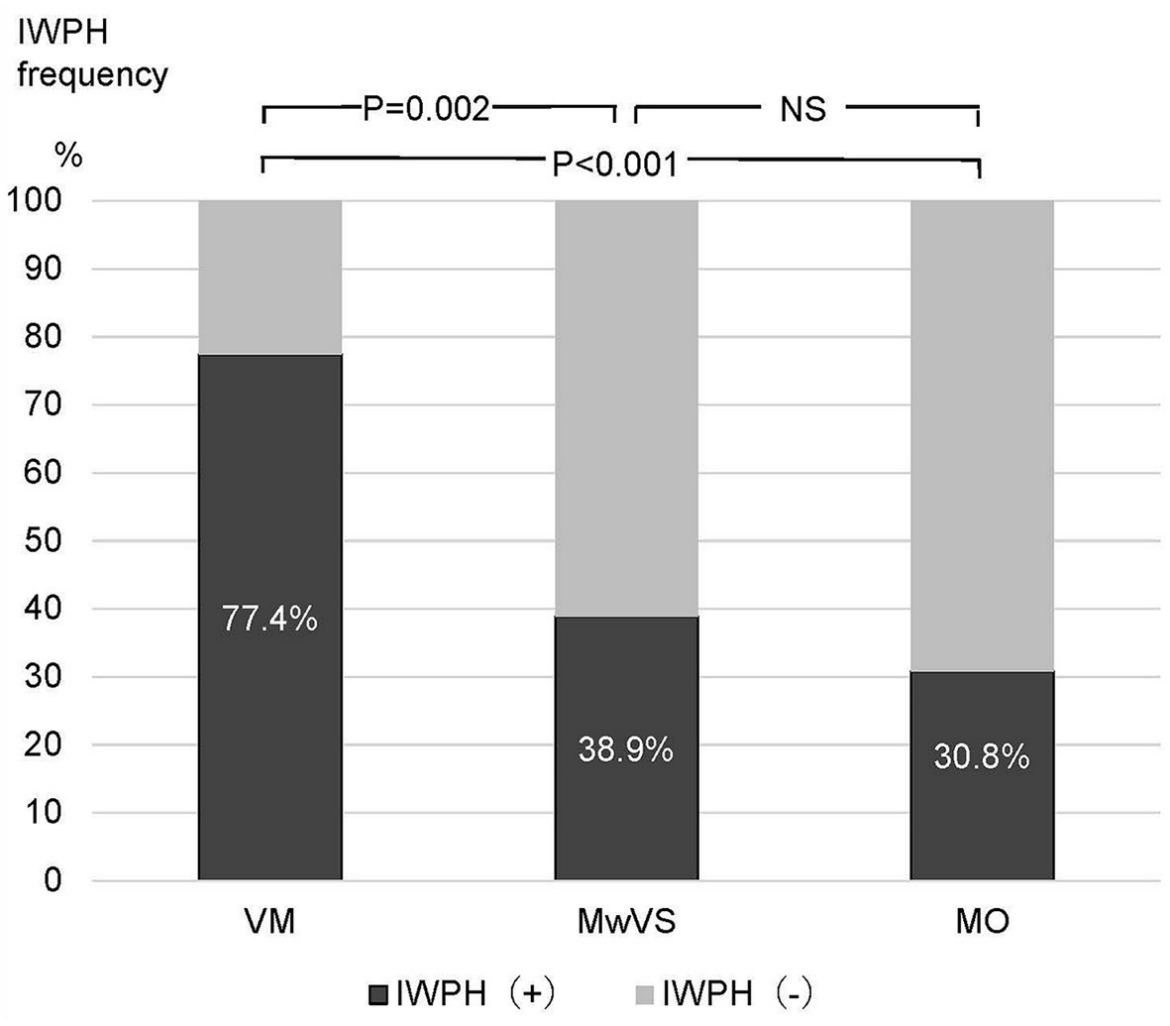
Comparison of interictal widespread pressure hyperalgesia frequency differences.

*Post-hoc* analysis revealed that IWPH was significantly more frequent in VM than in MwVS and MO (Figure 6). Logistic regression analysis confirmed IWPH as a significant determinant of VM. These findings support the notion that IWPH plays a crucial role in developing vestibular symptoms required for VM diagnosis. The pathophysiology of IWPH may involve impaired descending pain modulation (14, 33), which can amplify headache stimuli in the thalamus and induce thalamic sensitization. This sensitized thalamus may give rise to a widespread multimodal CA, possibly due to dysregulation of the descending pain modulation (33). Similar to the results of our previous study (17), no significant differences in CA subtypes were observed between VM and MwVS, including interictal CA. However, IWPH was significantly different between the two groups. This may be due to the suitability of hyperalgesia surveys over recall-based allodynia questionnaires in detecting interictal asymptomatic persistent central sensitization or sub-allodynia (12, 14, 55). As IWPH and acute CA were found to be correlated in our previous study (17), further investigation using QST during the headache-free phase may reveal differences in CA prevalence between VM and MwVS. The periaqueductal gray descending control selectively modulates C and Aδ nociceptive input (29). When compromised, amplified pain signals from the head, neck, and shoulders are transmitted to the thalamus via these fibers during headaches. Aβ fibers, not regulated by the descending system, transmit appropriate proprioceptive signals to the thalamus (56). This may disrupt the spatial integration of pain and proprioceptive signals in the thalamus and cortex, leading to dizziness. Our questionnaire survey revealed no significant difference in the prevalence of interictal CA, a symptom of persistent central sensitization, between VM and MwVS. However, a significant difference in the prevalence of IWPH between VM and MwVS was observed in the MTPS. This difference suggests varying levels of unperceived, persistent central sensitization between the two groups. Consequently, IWPH could act as a valuable clinical marker for differentiating VM from MwVS.

### Other features with no difference between VM and MwVS

#### Female sex

Despite previous reports suggesting that VM primarily affects females (7), our study found no significant sex-related difference between the VM, MwVS, and MO groups. While there is a potential female predominance in VM and MwVS compared to MO, this difference was not statistically significant (***p*** **=** 0.123). Further studies with larger sample sizes are needed to confirm these findings.

#### Headache intensity

The association between headache intensity and VM remains a topic of debate. Kutay et al. (45) found no significant difference in intensity between VM and migraines without vertigo, while others (38) have reported a strong correlation. The lack of significant differences in our study may be due to sampling bias favoring individuals with headache intensity ≥7.

#### HIT-6

In our previous study involving 143 interictal and 102 ictal migraine patients, we found that the HIT-6 score effectively differentiates VM from MwVS and MO (17). Thus, we concluded that the ICHD-IIIβ criteria for VM effectively identify severe cases of MwVS. However, we observed that the HIT-6 score was ineffective in identifying interictal migraine patients, possibly due to the small sample size.

#### Osmophobia

The prevalence of osmophobia among 85 patients with MwVS was 54.1% in the present study, similar to Akdal et al. (18, 36). Osmophobia was significantly more prevalent in the VM group than in the MO group, while photophobia and phonophobia did not differ significantly between groups. In this study, VM had a significantly higher prevalence of osmophobia than MO, in contrast to previous studies (17). This may be due to differences in interictally sustained central sensitization. Osmophobia is associated with allodynia (46), and further studies are needed to explore the relationship between interictal allodynia or interictal hyperalgesia and osmophobia.

#### Tinnitus

Tinnitus was observed in 45.2% of the VM group, consistent with previous studies (47–51). While the prevalence of tinnitus differed among the three groups, it was not significantly different between the VM and MwVS (*p* = 0.148, OR 2.84, 95% CI 1.00–8.34) based on *post-hoc* comparison. Consequently, tinnitus was included in the logistic regression model for further analysis.

#### Depression

There is a close interconnection between migraine, vestibular disorders, and psychological conditions such as anxiety and depression (45). Furman et al. have referred to this overlap as migraine–anxiety-related dizziness (39). In our previous studies, we observed variations in the prevalence of depression among the three groups (17), which were not evident in the current study. Specifically, the present findings revealed a lower prevalence of depression in the VM group (26%) compared to our previous report (34%). This discrepancy could be attributed to interictal anxiety being less severe than ictal anxiety, leading to lower SDS scores and less differentiation among the groups.

#### Sleep disorders

We did not observe a significant difference in the prevalence of sleep disorders among the three groups, which contradicts the findings of previous research (17, 52, 53). However, the prevalence of sleep disorders in the VM and MwVS groups (19% and 20%, respectively) was twice that of the MO group (10%). These findings suggest the possibility of potential differences that could be further elucidated with larger sample sizes could elucidate.

#### Allodynia

Consistent with the findings of our previous study (17), we observed significant differences in the prevalence of acute (*p* = 0.026), extracephalic (*p* = 0.008), mechanical (*p* = 0.01), and widespread multimodal CA (*p* = 0.006) among the three groups. However, cephalic (*p* = 0.052) and thermal (*p* = 0.688) CA did not differ significantly between the groups. Although a trend suggested a potential difference in the prevalence of cephalic CA among the three groups, further investigation is needed to confirm this. It is important to consider potential recall bias when evaluating the discomfort associated with heat stimuli (thermal CA) during headache attacks in the absence of headache. Further research is needed to examine this aspect more comprehensively. The prevalence of interictal CA was low with no significant differences among the groups (14%). Quantitative sensory testing (QST) may provide valuable insights into interictal CA. Additionally, a questionnaire-based investigation of widespread multimodal central sensitization, proposed as a clinical manifestation of thalami sensitization, revealed that both VM and MwVS exhibited equal levels of thalami sensitization, higher than MO. These findings suggest the potential involvement of thalamic sensitization in the pathophysiology of VM and MwVS. Moreover, our questionnaire assessment of allodynia in the absence of headaches indicated that VM and MwVS showed comparable levels of central sensitization compared to MO. To further explore this aspect, QST investigations in CA may provide insights into the potential association between VM and MwVS, regardless of the phase (acute or interictal), with unconscious CA (suballodynia).

### Candidate origin of vertigo during migraine

In light of our analysis, we propose that dizziness associated with VM can originate from four primary sources: (1) peripheral vertigo, linked to Meniere's disease-like disorders of the inner ear (57); (2) subcortical vertigo, stemming from altered vestibular and sub-allodynic input regulation by a sensitized thalamus (17); (3) cortical vertigo, potentially a focal symptom induced by CSD (44); or (4) vertigo caused by a compromised descending modulatory system, resulting in disrupted integration of perception within the thalamus and cortex.

### Strengths and limitations

This study's strengths include well-defined migraine statuses, comprehensive assessment of associated symptoms, and standardized semi-quantitative evaluation of IWPH. The prevalence of IWPH, an objective finding associated with central sensitization or dysfunction of the pain control system, was examined practically and reproducibly using the MTPS, which serves as a more accessible alternative to QST that requires specialized equipment and time. This is the first study to demonstrate that both aura and IWPH are significantly associated with VM compared to MwVS, facilitating differentiation between these conditions. By focusing on patients with migraine during headache-free intervals, the study identified clinical features that distinguish VM from MwVS. These findings contribute to a better understanding of VM and its distinct characteristics. However, our study has limitations that should be acknowledged. First, the recruiting of participants from a specialized headache clinic may have introduced sample bias, favoring those with moderate-to-intense headaches and moderate-to-less intense dizziness, potentially limiting the generalizability of our findings. Second, the use of a retrospective headache questionnaire may be susceptible to recall bias, especially when assessing symptoms such as allodynia and vestibular manifestations. Third, the reliance on a single rater for assessing IWPH may have influenced the inter-rater reliability. Fourth, our data-driven approach might blur confounder and risk distinctions. Future research should consider theory-driven models. Fifth, our inability to exclude migraine patients who may have coincidentally experienced five or more vertigo/dizziness episodes from other vestibular disorders and concurrent headaches. Finally, the cross-sectional design of our study only allows for observing associations between variables, and we cannot draw definitive conclusions about causal relationships between aura and VM or between IWPH and VM. Further research with longitudinal designs and larger, diverse samples is needed to address these limitations and provide more robust evidence in this area.

### Generalizability

The factors associated with VM in this study concern headache clinic patients, influenced by population variances, hospital referrals, and patients' preference for specialists. These factors are applicable specifically to patients seeking care at headache clinics and may not be representative of the general population. However, the demographic and clinical features of migraine, including the prevalence of vestibular symptoms, were consistent with findings from previous studies from various countries.

### Directions for future research

Further research is required to replicate the findings of this study in diverse populations and to investigate the relationship between vestibular symptoms, allodynia, and hyperalgesia using QST in conjunction with clinical examinations conducted by otorhinolaryngologists. The results of these clinical examinations may provide valuable insights into the pathophysiology of VM through the lens of central sensitization.

## Conclusions

In this cross-sectional study, we aimed to investigate the clinical characteristics, including IWPH as a potential marker of persistent central sensitization, among VM, MwVS, and MO in patients with interictal migraine. Our analysis revealed that aura and IWPH were more associated with VM than with MwVS and MO. No significant interictal differences were observed between MwVS and MO. Further, VM displayed a unique pathophysiology characterized by aura-related mechanisms and persistent central sensitization, particularly in relation to IWPH. These findings enhance our understanding of migraine variants, which may have implications for management strategies and the development of more targeted and effective treatments.

## Data availability statement

The original contributions presented in the study are included in the article/Supplementary material, further inquiries can be directed to the corresponding author.

## Ethics statement

The studies involving humans were approved by the Institutional Review Board of the Shinshu University School of Medicine (approval number 3552-1). The studies were conducted in accordance with the local legislation and institutional requirements. The participants provided their written informed consent to participate in this study. Written informed consent was obtained from the individual(s) for the publication of any potentially identifiable images or data included in this article.

## Author contributions

TT: Conceptualization, Data curation, Formal analysis, Investigation, Methodology, Project administration, Resources, Software, Visualization, Writing – original draft, Writing – review & editing. YH: Formal analysis, Investigation, Methodology, Validation, Writing – review & editing. TH: Conceptualization, Methodology, Project administration, Supervision, Validation, Writing – review & editing.

## References

[1] Neuhauser H Leopold M von Brevern M Arnold G Lempert T. The interrelations of migraine, vertigo, and migrainous vertigo. Neurology. (2001) 56:436–41. 10.1212/WNL.56.4.436 11222783

[2] Neuhauser HK. Chapter 5. The epidemiology of dizziness and vertigo. In:Furman JM Lempert T, editors. Handbook of Clinical Neurology. Amsterdam: Elsevier (2016). p. 67–82. 10.1016/B978-0-444-63437-5.00005-4 27638063

[3] Dieterich M Brandt T. Episodic vertigo related to migraine (90 cases): vestibular migraine? J Neurol. (1999) 246:883–92. 10.1007/s004150050478 10552234

[4] Iljazi A Ashina H Lipton RB Chaudhry B Al-Khazali HM Naples JG. Dizziness and vertigo during the prodromal phase and headache phase of migraine: a systematic review and meta-analysis. Cephalalgia. (2020) 40:1095–103. 10.1177/0333102420921855 32349538 PMC7483950

[5] Headache Classification Committee of the International Headache Society (I). The international classification of headache disorders^.^ 3rd ed. (beta version). Cephalalgia (2013) 33:629–808. 10.1177/0333102413485658 23771276

[6] Akdal G Ozge A Ergör G. The prevalence of vestibular symptoms in migraine or tension-type headache. J Vestib Res. (2013) 23:101–6. 10.3233/VES-130477 23788138

[7] Huang TC Wang SJ Kheradmand A. Vestibular migraine: an update on current understanding and future directions. Cephalalgia. (2020) 40:107–21. 10.1177/0333102419869317 31394919

[8] Bednarczuk NF Bonsu A Ortega MC Fluri AS Chan J Rust H. Abnormal visuo-vestibular interactions in vestibular migraine: a cross sectional study. Brain. (2019) 142:606–16. 10.1093/brain/awy355 30759189 PMC6391603

[9] Jeong SH Oh SY Kim HJ Koo JW Kim JS. Vestibular dysfunction in migraine: effects of associated vertigo and motion sickness. J Neurol. (2010) 257:905–12. 10.1007/s00415-009-5435-5 20041331

[10] Burstein R Yarnitsky D Goor-Aryeh I Ransil BJ Bajwa ZH. An association between migraine and cutaneous allodynia. Ann Neurol. (2000) 47:614–24. 10.1002/1531-8249(200005)47:5&lt;614::AID-ANA9&gt;3.0.CO;2-N 10805332

[11] Nicolodi M Sicuteri R Coppola G Greco E Pietrini U Sicuteri F. Visceral pain threshold is deeply lowered far from the head in migraine. Headache. (1994) 34:12–9. 10.1111/j.1526-4610.1994.hed3401012.x 8132435

[12] Weissman-Fogel I Sprecher E Granovsky Y Yarnitsky D. Repeated noxious stimulation of the skin enhances cutaneous pain perception of migraine patients in-between attacks: clinical evidence for continuous sub-threshold increase in membrane excitability of central trigeminovascular neurons. Pain. (2003) 104:693–700. 10.1016/S0304-3959(03)00159-3 12927642

[13] Louter MA Bosker JE van Oosterhout WPJ van Zwet EW Zitman FG Ferrari MD. Cutaneous allodynia as a predictor of migraine chronification. Brain. (2013) 136:3489–96. 10.1093/brain/awt251 24080152

[14] Ashkenazi A Silberstein S Jakubowski M Burstein R. Improved identification of allodynic migraine patients using a questionnaire. Cephalalgia. (2007) 27:325–9. 10.1111/j.1468-2982.2007.01291.x 17376108 PMC2664545

[15] Jakubowski M Silberstein S Ashkenazi A Burstein R. Can allodynic migraine patients be identified interictally using a questionnaire? Neurology. (2005) 65:1419–22. 10.1212/01.wnl.0000183358.53939.38 16275830

[16] Bernstein C Burstein R. Sensitization of the trigeminovascular pathway: perspective and implications to migraine pathophysiology. J Clin Neurol. (2012) 8:89–99. 10.3988/jcn.2012.8.2.89 22787491 PMC3391624

[17] Toriyama T Hanaoka Y Horiuchi T. Clinical features of definite vestibular migraine through the lens of central sensitization: a cross-sectional study. Acta Neurol Belg. (2022) 122:1511–9. 10.1007/s13760-021-01772-5 34370217

[18] Akdal G Baykan B Ertaş M Zarifoglu M Karli N Saip S. Population-based study of vestibular symptoms in migraineurs. Acta Otolaryngol. (2015) 135:435–9. 10.3109/00016489.2014.969382 25662067

[19] Abouzari M Goshtasbi K Moshtaghi O Tan D Lin HW Djalilian HR. Association between vestibular migraine and migraine headache: yet to explore. Otol Neurotol. (2020) 41:392–6. 10.1097/MAO.0000000000002528 31821258 PMC8040771

[20] Scholten-Peeters GGM Coppieters MW Durge TSC Castien RF. Fluctuations in local and widespread mechanical sensitivity throughout the migraine cycle: a prospective longitudinal study. J Headache Pain. (2020) 21:16. 10.1186/s10194-020-1083-z 32059650 PMC7023769

[21] Özçelik P Koçoglu K Öztürk V Keskinoglu P Akdal G. Characteristic differences between vestibular migraine and migraine only patients. 269(1):336-341. 10.1007/s00415-021-10636-0 34109480

[22] Filatova E Latysheva N Kurenkov A. Evidence of persistent central sensitization in chronic headaches: a multi-method study. J Headache Pain. (2008) 9:295–300. 10.1007/s10194-008-0061-7 18690491 PMC3452198

[23] Lipton RB Bigal ME Ashina S Burstein R Silberstein S Reed ML. Cutaneous allodynia in the migraine population. Ann Neurol. (2008) 63:148–58. 10.1002/ana.21211 18059010 PMC2729495

[24] Okifuji A Turk DC Sinclair JD Starz TW Marcus DA. A standardized Manual Tender Point Survey I Development and determination of a threshold point for the identification of positive tender points in fibromyalgia syndrome. J Rheumatol. (1997) 24:377–83. 9035000

[25] Lempert T Olesen J Furman J Waterston J Seemungal B Carey J. Vestibular migraine: diagnostic criteria. J Vestib Res. (2012) 22:167–72. 10.3233/VES-2012-0453 23142830

[26] Chanques G Viel E Constantin JM Jung B de Lattre S Carr J. The measurement of pain in intensive care unit: comparison of 5 self-report intensity scales. Pain. (2010) 151:711–21. 10.1016/j.pain.2010.08.039 20843604

[27] Kosinski M Bayliss MS Bjorner JB Ware JE Garber WH Batenhorst A. A six-item short-form survey for measuring headache impact: the HIT-6TM. Qual Life Res. (2003) 12:963–74. 10.1023/A:1026119331193 14651415

[28] Zung WWK. A self-rating depression scale. Arch Gen Psychiatry. (1965) 12:63–70. 10.1001/archpsyc.1965.01720310065008 14221692

[29] Guy N Marques AR Orliaguet T Lanteri-Minet M Dallel R Clavelou P. Are there differences between cephalic and extracephalic cutaneous allodynia in migraine patients? Cephalalgia. (2010) 30:881–6. 10.1111/j.1468-2982.2009.02008.x 19740124

[30] Lovati C D'Amico D Bertora P Rosa S Suardelli M Mailland E. Acute and interictal allodynia in patients with different headache forms: an Italian pilot study. Headache. (2008) 48:272–7. 10.1111/j.1526-4610.2007.00998.x 18081821

[31] Burstein R Jakubowski M Garcia-Nicas E Kainz V Bajwa Z Hargreaves R. Thalamic sensitization transforms localized pain into widespread allodynia. Ann Neurol. (2010) 68:81–91. 10.1002/ana.21994 20582997 PMC2930514

[32] Wolfe F Smythe HA Yunus MB Bennett RM Bombardier C Goldenberg DL. The American College of Rheumatology 1990 criteria for the classification of fibromyalgia. Report of the multicenter criteria committee. Arthritis Rheum. (1990) 33:160–72. 10.1002/art.1780330203 2306288

[33] Toriyama T Horiuchi T Hongo K. Characterization of migraineurs presenting interictal widespread pressure hyperalgesia identified using a tender point count: a cross-sectional study. J Headache Pain. (2017) 18:117. 10.1186/s10194-017-0824-0 29285568 PMC5745372

[34] Kanda Y. Investigation of the freely available easy-to-use software “EZR” for medical statistics. Bone Marrow Transplant. (2013) 48:452–8. 10.1038/bmt.2012.244 23208313 PMC3590441

[35] Kayan A Hood JD. Neuro-otological manifestations of migraine. Brain. (1984) 107:1123–42. 10.1093/brain/107.4.1123 6334543

[36] Zhang Y Kong Q Chen J Li L Wang D Zhou J. International Classification of Headache Disorders 3rd edition beta-based field testing of vestibular migraine in China: demographic, clinical characteristics, audiometric findings and diagnosis statues. Cephalalgia. (2016) 36:240–8. 10.1177/0333102415587704 25986149

[37] Cho SJ Kim BK Kim BS Kim JM Kim SK Moon HS. Vestibular migraine in multicenter neurology clinics according to the appendix criteria in the third beta edition of the International Classification of Headache Disorders. Cephalalgia. (2016) 36:454–62. 10.1177/0333102415597890 26224714

[38] Calhoun AH Ford S Pruitt AP Fisher KG. The point prevalence of dizziness or vertigo in migraine—and factors that influence presentation. Headache. (2011) 51:1388–92. 10.1111/j.1526-4610.2011.01970.x 21797862

[39] Furman JM Marcus DA Balaban CD. Vestibular migraine: clinical aspects and pathophysiology. Lancet Neurol. (2013) 12:706–15. 10.1016/S1474-4422(13)70107-8 23769597

[40] Johnson GD. Medical management of migraine-related dizziness and vertigo. Laryngoscope. (1998) 108:1–28. 10.1097/00005537-199801001-00001 9430502

[41] Stolte B Holle D Naegel S Diener HC Obermann M. Vestibular migraine. Cephalalgia. (2015) 35:262–70. 10.1177/0333102414535113 24847169

[42] Zorzin L Carvalho GF Kreitewolf J Teggi R Pinheiro CF Moreira JR. Subdiagnosis, but not presence of vestibular symptoms, predicts balance impairment in migraine patients – a cross sectional study. J Headache Pain. (2020) 21:56. 10.1186/s10194-020-01128-z 32448118 PMC7247141

[43] Cutrer FM Baloh RW. Migraine-associated dizziness. Headache. (1992) 32:300–4. 10.1111/j.1526-4610.1992.hed3206300.x 1399552

[44] Demarquay G Ducros A Montavont A Mauguiere F. Migraine with brainstem aura: why not a cortical origin? Cephalalgia. (2018) 38:1687–95. 10.1177/0333102417738251 29073774

[45] Kutay Ö Akdal G Keskinoglu P Balci BD Alkin T. Vestibular migraine patients are more anxious than migraine patients without vestibular symptoms. J Neurol. (2017) 264:37–41. 10.1007/s00415-017-8439-6 28280987

[46] Delussi M Laporta A Fraccalvieri I de Tommaso M. Osmophobia in primary headache patients: associated symptoms and response to preventive treatments. J Headache Pain. (2021) 22:109. 10.1186/s10194-021-01327-2 34537019 PMC8449918

[47] Power L Shute W Mcowan B Murray K Szmulewicz D. Clinical characteristics and treatment choice in vestibular migraine. J Clin Neurosci. (2018) 52:50–3. 10.1016/j.jocn.2018.02.020 29550250

[48] Neff BA Staab JP Eggers SD Carlson ML Schmitt WR Van Abel KM. Auditory and vestibular symptoms and chronic subjective dizziness in patients with Ménière's disease, vestibular migraine, and Ménière's disease with concomitant vestibular migraine. Otol Neurotol. (2012) 33:1235–44. 10.1097/MAO.0b013e31825d644a 22801040

[49] Lopez-Escamez JA Dlugaiczyk J Jacobs J Lempert T Teggi R von Brevern M. Accompanying symptoms overlap during attacks in Menière's disease and vestibular migraine. Front Neurol. (2014) 5:265. 10.3389/fneur.2014.00265 25566172 PMC4265699

[50] Morganti LOG Salmito MC Duarte JA Bezerra KC Simões JC Ganança FF. Vestibular migraine: clinical and epidemiological aspects. Braz J Otorhinolaryngol. (2016) 82:397–402. 10.1016/j.bjorl.2015.06.003 26614042 PMC9449005

[51] Neuhauser HK Radtke A von Brevern M Feldmann M Lezius F Ziese T. Migrainous vertigo: prevalence and impact on quality of life. Neurology. (2006) 67:1028–33. 10.1212/01.wnl.0000237539.09942.06 17000973

[52] Salhofer S Lieba-Samal D Freydl E Bartl S Wiest G Wöber C. Migraine and vertigo—a prospective diary study. Cephalalgia. (2010) 30:821–8. 10.1177/0333102409360676 20647173

[53] Wu J Liu C Yu H Li H Jia Y Zhang D. Clinical characteristics of sleep disorders in patients with vestibular migraine. Sleep Breath. (2020) 24:1383–8. 10.1007/s11325-019-01994-1 31832981

[54] Carli G Suman AL Biasi G Marcolongo R. Reactivity to superficial and deep stimuli in patients with chronic musculoskeletal pain. Pain. (2002) 100:259–69. 10.1016/S0304-3959(02)00297-X 12467997

[55] Sand T Zhitniy N Nilsen KB Helde G Hagen K Stovner LJ. Thermal pain thresholds are decreased in the migraine preattack phase. Eur J Neurol. (2008) 15:1199–205. 10.1111/j.1468-1331.2008.02276.x 18795945

[56] Haggard P Iannetti GD Longo MR. Spatial sensory organization and body representation in pain perception. Curr Biol. (2013) 23:R164–76. 10.1016/j.cub.2013.01.047 23428330

[57] Burstein R Jakubowski M Rauch SD. The science of migraine. J Vestib Res. (2011) 21:305–14. 10.3233/VES-2012-0433 22348935 PMC3690498

